# The peripheral immune response after stroke—A double edge sword for blood‐brain barrier integrity

**DOI:** 10.1111/cns.13081

**Published:** 2018-11-01

**Authors:** Yan Li, Zi‐Yu Zhu, Ting‐Ting Huang, Yu‐Xi Zhou, Xin Wang, Li‐Qun Yang, Zeng‐Ai Chen, Wei‐Feng Yu, Pei‐Ying Li

**Affiliations:** ^1^ Department of Anesthesiology, Renji Hospital, School of Medicine Shanghai Jiaotong University Shanghai China; ^2^ Department of Radiology, Renji Hospital, School of Medicine Shanghai Jiaotong University Shanghai China

**Keywords:** blood‐brain barrier, peripheral immune response, stroke

## Abstract

The blood‐brain barrier (BBB) is a highly regulated interface that separates the peripheral circulation and the brain. It plays a vital role in regulating the trafficking of solutes, fluid, and cells at the blood‐brain interface and maintaining the homeostasis of brain microenvironment for normal neuronal activity. Growing evidence has led to the realization that ischemic stroke elicits profound immune responses in the circulation and the activation of multiple subsets of immune cells, which in turn affect both the early disruption and the later repair of the BBB after stroke. Distinct phenotypes or subsets of peripheral immune cells along with diverse intracellular mechanisms contribute to the dynamic changes of BBB integrity after stroke. This review focuses on the interaction between the peripheral immune cells and the BBB after ischemic stroke. Understanding their reciprocal interaction may generate new directions for stroke research and may also drive the innovation of easy accessible immune modulatory treatment strategies targeting BBB in the pursuit of better stroke recovery.

## 1 INTRODUCTION

Ischemic stroke is one of the most common neurological disorders and a major cause of disability and death with limited transitional success of mounting stroke researches, posing an economic and societal burden.^1^, ^2^ Under normal condition, the brain is under continuous immune surveillance and regulation. The neurovascular unit (NVU) regulates the homeostasis of brain microenvironment for normal neuronal activity. It is composed of neurons, glial cells (oligodendrocytes, microglia, and astrocytes) and vascular cells (endothelial cells, pericytes, and smooth muscle cells as well as the basal lamina matrix within brain vasculature).^3^, ^4^ Compared to the concept of NVU, the notion of blood‐brain barrier (BBB), which traditionally includes endothelia cells (ECs), astrocytes, pericytes, and the basal lamina matrix, tight junction proteins within the vasculature is more straight forward and more intensively studied.^5^, ^6^, ^7^, ^8^, ^9^, ^10^, ^11^, ^12^

BBB damage is a common pathological feature shared by stroke and a variety of neurological diseases.^13^, ^14^, ^15^, ^16^, ^17^, ^18^ Notably, it is closely associated with poor neurological outcomes.^19^, ^20^, ^21^, ^22^, ^23^ In response to cerebral ischemic stroke, the brain can release a variety of “danger” signals or “help me” signals, such as ATP, high‐mobility group box 1 (HMGB1), hypoxia‐inducible factor 1α (HIF‐1α), S100B, brain‐derived antigens and et al, all of which activate multiple subsets of peripheral immune cells^24^, ^25^.Once activated, these cells can migrate to the ischemic brain through detection of chemoattractant gradients.^26^, ^27^, ^28^ Upon BBB disruption, the components in blood including immune cells can enter into brain sequentially and interaction of neuro‐immune interaction can be initiated.^29^, ^30^, ^31^, ^32^ During their penetration through the injured BBB, these immune cells become a double edge sword, which could either exacerbate the BBB disruption or protect the integrity of BBB.^36^, ^37^ Pleiotropic intracellular mechanisms and diverse secretory factors, including cytokines, proteolytic enzymes, exosomes, micro vesicles, and miRNAs, have been implicated in their interaction both in early disruption and later repair phase of the BBB.^33^, ^34^, ^35^, ^36^, ^37^ Importantly, distinct phenotypes and subsets of immune cells exhibit diverse impact on the poststroke BBB. This review will discuss the dynamic changes of BBB integrity after stroke followed by a discussion of the double‐faced roles of peripheral immune activation on the BBB integrity after stroke. Finally, the newly emerged mechanisms by which the peripheral immune cells impact the BBB integrity have also been covered at the end, including exosomes and micro vesicles.

## 2 DYNAMIC CHANGES OF BLOOD‐BRAIN BARRIER INTEGRITY (BBB) AFTER STROKE

In response to ischemic stroke, the integrity of BBB compromises promptly and changes dynamically, which can be observed by magnetic resonance imaging (MRI) and other imaging techniques.^38^, ^39^, ^40^, ^41^, ^42^ During the early phase, cytoskeletal alterations in brain ECs can be initiated by actin polymerization followed by translocation of tight junctions (TJs) within 30‐60 minutes of reperfusion through activation of the Rho‐associated protein kinase (ROCK)/myosin.^43^ The activation of ROCK pathway may further promote the cross‐linking of F‐actin into force‐generating linear stress fibers through the phosphorylation/activation of myosin light chains (MLC) and increase cytoskeletal tension thus lead to the disassembly of TJs. These changes can widen the paracellular space between ECs, eventually resulting in hyperpermeability.^43^ In addition, opening of sodium and calcium channels, endothelial connexin‐43 hemichannels, the alterations in endocytotic vesicles, endothelial endocytosis/transcytosis, and transcellular vesicular trafficking, which could be mediated by caveolin‐1, endothelial growth factor, or exocytotic machinery, may also account for BBB hyperpermeability as early as 6 hours after cerebral stroke.^44^, ^45^, ^46^, ^47^ Increased expression of aquaporin 4 (AQP4) in astrocytes of the ischemic brain is also associated with the initial cerebral edema after stroke.^48^, ^49^, ^50^ Additionally, pericytes may separate from basement membrane and detach from endothelial cells through paracellular pathway or transcellular routes, which contributes to increased microvascular permeability via disruption of pericyte‐tight junction interactions.^51^, ^52^, ^53^

The expression of TJ proteins may be decreased due to increased oxidative stress or matrix metalloproteinases 9 (MMP9)‐mediated protein degradation after stroke, leading to a “second wave” of increased BBB permeability.^40^ Disruption of BBB integrity is not only a common consequence of stroke, but also contributes to the progression of stroke.^54^, ^55^ Infarct size can progress with time after cerebral ischemia reperfusion, with more resident immune cells activated and more peripheral immune cells recruited to the brain and thereby further aggravates the injury of BBB.^56^ Cerebral ischemia upregulates the expression of adhesion molecules, such as intercellular adhesion molecule 1 (ICAM‐1), vascular cell adhesion molecule 1 (VCAM‐1), integrin and E‐selectin in the brain, which in turn facilitate a massive “second wave” of immune cell entry into the brain parenchyma through the BBB, leading to exacerbated neuroinflammation. The inflammation in the lesioned brain also contributes to the “second wave” of BBB disruption.^57^, ^58^ At 1‐3 days after stroke, BBB breakdown is featured by TJ degradation, basement membrane disruption, and eventually loss of endothelial cells.^59^ Chronic BBB opening caused by the loss of pericytes or chronic stress can lead to neuronal uptake of multiple blood‐derived neurotoxic products as well as reductions in microcirculation that in turn results in a chronic neuronal dysfunction and degenerative changes.^60^, ^61^

**Figure 1:**
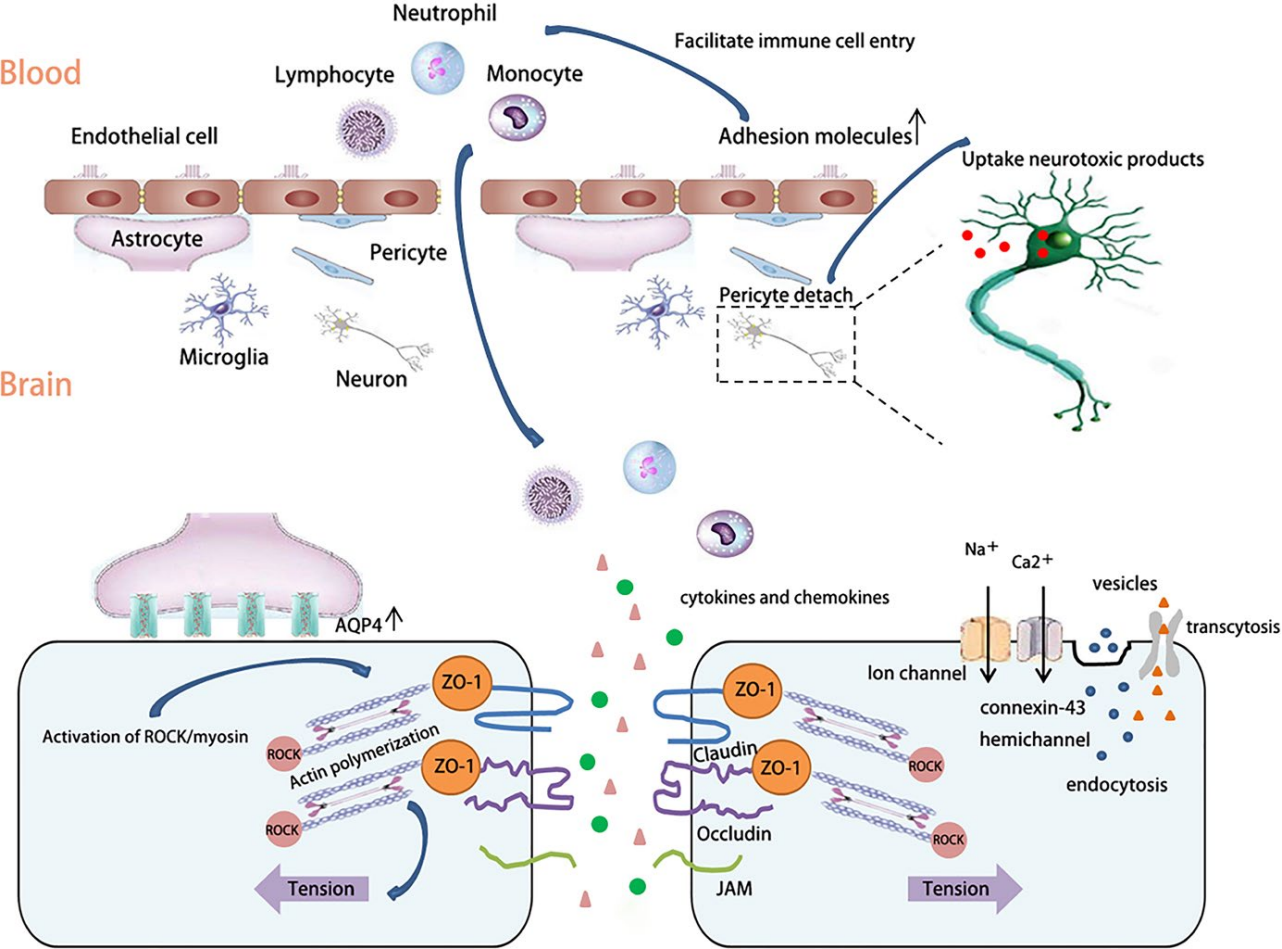
Blood‐brain barrier (BBB) integrity changes dynamically after ischemic stroke, BBB is composed of four major components, endothelia cells (ECs), basement membrane, astrocytes, and pericytes. After ischemic stroke, the structural of these cells changed. A, ECs: ECs are basic components and mainly connected by TJs to control the permeability of BBB. TJs comprise of junction adhesion molecules (JAM), claudins, and occludins, all of which are linked to the cytoskeleton via zonula occludens (ZO) protein. After ischemic stroke, cytoskeletal alterations in brain ECs are initiated by actin polymerization and increased cytoskeletal tension. Increased endocytosis/transcytosis along with opening of ion channels and endothelial connexin‐43 hemichannels on ECs also contribute to brain edema. Increased expression of adhesion molecules attracts peripheral immune cells to enter the brain and release immune factors. B, Pericytes: pericytes detach from ECs, widening paracellular spaces. Neurotoxic products influx into neurons, causing neuronal injury. C, Astrocytes: the expression of AQP4 water channels elevates on astrocytes and leads to brain edema. All of the above changes contribute to the disruption of BBB after stroke

During the late phase of stroke, the BBB dysfunction becomes less severe, in which BBB restoration possibly plays an important role.^62^ Multiple processes may be involved in the restoration of BBB permeability after stroke at the late phase of stroke. Neovascularization begins in the peri‐infarct region, which involves the proliferation of endothelial cells and sprouting of the vessels that eventually increase vascular density.^24^ Astrocytes act to maintain endothelial permeability and survival after stroke through improving tight junction constituents.^52^, ^63^ Pericytes can stable actin filaments in endothelial cells and preventing their death, guiding the correct location of endothelial cells, and clearing up neuronal debris during injury.^52^, ^64^ The illustration of structural changes of ECs, TJs, astrocytes, and pericytes of the BBB after ischemic stroke is shown in Figure 1.

## 3 THE DOUBLE‐FACED ROLES OF PERIPHERAL IMMUNE ACTIVATION ON THE BBB INTEGRITY AFTER STROKE

The injured ischemic brain can release ATP, high‐mobility group box 1 (HMGB1), HIF‐1α, S100B, brain‐derived antigens and et al as alarm signals to activate the peripheral immune system through purinergic receptors, TLRs, and the receptor for advanced glycation endproducts (RAGE).^61^ Once activated, these cells can migrate to areas of injury through detection of chemoattractant gradients.^24^, ^26^ During their penetration through the injured BBB, these immune cells become a double edge sword, which could either exacerbate the BBB disruption or protect the integrity of BBB.

### 3.1 Activated PMNS and BBB disruption after ischemic stroke

Polymorphonuclear neutrophils (PMNs) are the most abundant cell population present at the site of injury with a peak influx between 1 and 3 days in the ischemic brain.^56^, ^65^, ^66^ Infiltration of PMNs is closely related to BBB disruption by a series of biological processes, such as releasing proteases, MMP, elastase, cathepsin G, and proteinase, producing reactive oxygen species (ROS), causing endothelial dysfunction and disorganization of junctional proteins, all of which are known to damage the BBB.^67^, ^68^

MMP9 is a subtype of matrix metalloproteinase. It may completely degrade the basal lamina and TJ components through attacking type IV collagen, laminin, and fibronectin and result in gross barrier disruption,^69^, ^70^ brain edema, leukocyte infiltration, and hemorrhage.^71^, ^72^, ^73^ Under normal condition, the expression of MMPs in the brain is very low. Ischemic stroke may induce increased expression of MMPs, especially MMP9.^74^ Both brain vascular ECs and infiltrating neutrophils can produce MMP9 after focal cerebral ischemia, which can be used to predict stroke patient outcome.^74^, ^75^, ^76^, ^77^ In ischemic stroke patients, MMP9 increases in the plasma within the first 2 to 6 hours and the MMP9 mRNA in leukocytes increased within 3 hours^78^ Activated neutrophils may be an important source of pre‐existing intracellular MMP9 pool, which can be secreted in response to middle cerebral artery occlusion (MCAO) and oxygen‐glucose deprivation (OGD) insults^69^ In addition, activated MMPs may also be triggered by increased TNF‐*α, *IL‐6, and α2‐antiplasmin in the blood^79^, ^80^ The granulocyte‐colony stimulating factor (G‐CSF) can stimulate the proliferation of peripheral neutrophils and thus increase the level of MMP9, leading to exacerbated BBB disruption and increased ischemic infarction.^81^

In addition to MMP9,the elastase secreted by neutrophils also degrades basal lamina and extracellular matrix.^82^ In mice lacking elastase, ischemia‐induced BBB disruption is reduced, as is infarct volume and cerebral edema. Inhibiting elastase can further decrease infarct volume and BBB disruption in MMP9‐null mice, suggesting an MMP9 independent mechanism of elastase on the BBB disruption.^83^ Besides MMP9 and elastase, neutrophils are important source of ROS in cerebral ischemia and reperfusion injury. ROS itself can disrupt the BBB through direct damage to endothelial cells, pericytes, smooth muscle cells, and astrocytes.^84^ In addition, activated PMNs stimulate inflammatory cytokine production, which attracts more leukocytes from the periphery and aggravates the adhesion molecule expression on ECs, thus further propagates the postischemic inflammation cascade that exacerbates BBB disruption^85^ and increases the risk of secondary bleeding within the ischemic focus.^46^, ^71^, ^86^

### 3.2 The bright side of PMNS in poststroke BBB disruption

Given the well‐established negative impact of poststroke BBB disruption, PMNs may also have beneficial effects for the BBB to repair in the late phase of stroke.^87^ MMP9, which induces BBB disruption in the early phase, is suggested to promote BBB remodeling in the late phase of stroke by enhancing degradation of proinflammatory DAMPs and vascular remodeling.^87^, ^88^, ^89^, ^90^ In addition, PMNs can release antiinflammatory molecules, such as annexin‐1, lipoxin A4, resolvins, and protectins to alleviate the poststroke inflammatory reaction.^91^ PMNs also attract monocytes that clear apoptotic neutrophils and cellular debris through phagocytosis.^36^ Neutrophil‐derived MMP9 is also involved in the regulation of the proangiogenic and release hematopoietic progenitor cells from the bone marrow.^89^ Like monocytes, PMNs are divided into two phenotypes, N1 and N2 phenotype. The N2 phenotype is encompassed with antiinflammatory properties that may have protective effects in stroke.^92^ Thus, under certain conditions neutrophils are not detrimental and may be beneficial in the progression of stroke.

### 3.3 Pleiotropic effects of microglia/macrophages on the BBB integrity after stroke

The function of microglia/macrophages function is complex and largely depends on the existence of varied, plastic, and multilayered macrophage phenotypes.^93^ The impact of microglia/macrophages activation on the ischemic BBB largely depends on the phenotype or status of these cells which can be affected by micro‐environmental cues.^94^, ^95^, ^96^ According to distinct cues, the microglia/macrophages can differentiate into two subtypes—inflammatory and antiinflammatory phenotypes.^97^ In their proinflammatory phenotypes, they can aggravate the BBB injury while in their phagocytosis or antiinflammatory phenotypes they may play distinct roles toward BBB repair and regeneration.^98^, ^99^, ^100^, ^101^ However, recent studies suggest that the terminology of microglia/macrophage polarization may be limited. Transcriptomic and proteomic profiles, regional heterogeneity, sexual dimorphism, and age could all take into account while determining the functions of microglia/macrophages.^102^

The destructive effects on BBB are mainly mediated by M1 phenotypes which are characterized by the production of proinflammatory mediators including IL‐1β, TNF‐α, IL‐6, and IL‐12 and MHC II,^103^, ^104^ MCP1/CCL2.^105^, ^106^ These effects mainly fall into several categories: (a) increased expression of inducible nitric oxide synthase (iNOS); (b) increased production of ROS; (c) synthesis of proteolytic enzymes (MMP9, MMP3)^107^; (d) upregulated expression of macrophage migration inhibitory factor (MIF); (v) phagocytosis of endothelial cells; and (vi) recruit other proinflammatory cells to further exacerbate the inflammatory cascade. It has been reported that proinflammatory cytokines together with nitric oxide (NO) and proteolytic enzymes can induce the increase of BBB permeability^108^ by downregulating TJ proteins expression in ECs and modulate the expression of adhesion molecules.^109^ ROS, generated by NADPH oxidase, provokes EC contractions and consequently increases permeability of the BBB.^110^ Both inflammatory microglia and macrophages have been suggested to produce ROS after neurological diseases, including stroke.^111^, ^112^, ^113^, ^114^ MIF, also known as glycosylation‐inhibiting factor (GIF) is an important regulator of innate immunity. It has been shown to promote leukocyte‐endothelial cell interactions through promoting endothelial adhesion molecule expression.^115^, ^116^ It has recently been suggested to directly degrade the BBB after ischemic brain.^117^ Perivascular microglia/macrophages migrate toward the disrupted blood vessels and further damage them by phagocytizing ECs.^118^ Simultaneously, they also attract more proinflammatory cells, such as Th1 cells by secreting CXCL9, CXCL10 and IL‐6 and IL‐23.^119^, ^120^, ^121^, ^122^, ^123^ The interaction between microglia/macrophages and monocytes and lymphocytes may further exacerbate the BBB damage and immune cascades.^110^

Unlike the M1 phenotype, microglia/macrophages have a distinct M2 phenotype, which is mainly protective in cerebral ischemic injury.^124^, ^125^ The impact of M2 microglia/macrophage on the BBB after stroke may include (a) immunosuppressive functions; (b) phagocytosis of ischemic debris; and (c) pro‐angiogenesis. Initially, M2 are the dominant cell type in the ipsilateral penumbra after stroke.^126^ They can not only express antiinflammatory cytokines, such as IL‐4, IL‐10, and TGF‐β by themselves to maintain the integrity of the neurovascular unit in murine stroke models^99^, ^127^, ^128^, ^129^ but also stimulate Th2 cells, which produce high levels of IL10 and IL13^130^ and drive Treg polarization by IL‐10 and TGF‐β.^131^ Both microglia and infiltrated macrophages can migrate into the infarction area and elicited phagocytic response, which contribute to the clearance of cell debris or hematoma in the context of ischemia and intracerebral hemorrhage.^118^, ^132^ Osteopontin (OPN), an adhesive glycoprotein,^133^ has been suggested as a cell surface receptor associated with their phagocytosis function after ischemic stroke.^133^ Phagocytosis by microglia/macrophages can also exert favorable effects through engulfment of infiltrated PMN.^36^

### 3.4 Dualistic roles of T lymphocytes on the BBB integrity after stroke

Similar to neutrophils and microglia/macrophages, peripheral T lymphocytes infiltrated to the ischemic brain also exert dualistic roles on the evolution of BBB damage based on their different subtypes.

T‐helper 1(Th1), Th17, γδT cells, and CD8^+^ T cells mainly play detrimental roles on the BBB disruption after several neurological diseases, including stroke.^84^, ^99^, ^134^, ^135^, ^136^ Th1 cells promote BBB permeability by secreting proinflammatory cytokines (IL‐2, IFN‐γ, and TNF‐α) and mediating a cellular immune response.^137^ IFN‐γ activates the small GTPase RhoA and increases the expression of Rho‐associated kinase (ROCK), which in turn phosphorylates and activates MLC.^138^ TNF‐α stimulates NF‐kβ to increase myosin light chain kinase (MLCK) transcription, which further correlates with increased MLCK protein levels, MLC hyper‐phosphorylation, and paracellular permeability. Activated MLCK phosphorylates MLC and decreases TJ protein amounts, leading to cytoskeletal rearrangement and impairment of TJ integrity.^139^ In addition, Th1 cells can interact with M1 phenotype through releasing soluble cytokines, which transform the microglia to M1 type and thereby increase secondary ischemic damage.^140^ Th17 cells release IL‐17, IL‐21, and IL‐22^34^ and clear pathogens by inflammatory immunity,^141^ playing a proinflammatory role distinguished from Th1 cells. Th17 cells are demonstrated to disrupt the BBB by the activation of IL‐17A and promote the recruitment of additional CD4^+^ lymphocytes.^84^ IL‐17A induced NADPH oxidase‐dependent ROS production. The resulting oxidative stress activated the endothelial contractile machinery, which was accompanied by a downregulation of the TJ molecule occluding.^84^, ^142^ As unconventional T lymphocytes, γδT cells respond swiftly to ischemia and are regarded as detrimental to the BBB, largely through their production of cytotoxic cytokines, including IL‐17.^136^, ^143^ Depletion of γδT cells reduces brain injury secondary to experimental stroke with reperfusion.^144^ CD8^+^ T cells mainly promote BBB damage and play proinflammatory roles by killing target cells directly or indirectly.^144^ They initiate BBB breakdown through perforin‐mediated disruption of TJs. In turn, leakage from the vasculature into the parenchyma causes brain swelling and edema.^145^, ^146^

The subtype of Th2 cells mainly exerts antiinflammatory function thus maintain the BBB integrity after stroke by releasing antiinflammatory cytokines, such as IL‐4, IL‐5, IL‐10, and IL‐13, which can promote the M2 polarization.^120^, ^147^, ^148^ Regulatory T cells (Tregs) are one of the most important subtypes of Th2 cells in protecting ischemic brain injury. They release TGF‐β and IL‐10^149^ to maintain immune tolerance and counteract tissue damage.^150^ They inhibit the activation of neutrophils, lymphocytes, and microglia, thus function as key endogenous modulators of postischemic neuroinflammation.^151^ The inhibition of neutrophils of Tregs through the inhibitory molecule programmed death‐ligand 1 significantly reduces the level of MMP9, thus protects against BBB disruption after stroke and attenuates tPA‐induced hemorrhagic transformation.^152^, ^153^, ^154^ Importantly, adoptive transfer of Tregs does not exacerbate poststroke immunosuppression but improves immune status after focal cerebral ischemia^155^ and is beneficial for protection/repair following stroke.^131^ However, there are also conflicting data showing that depletion of Treg in a depletion of regulatory T cell(DEREG) mouse model protects brain from acute ischemic stroke while adoptive transfer of Tregs worsens outcome after ischemic stroke.^144^, ^150^, ^156^ The conflict finding may be attributed to discrepancies in Treg delivery protocols used in different studies.^157^

### 3.5 The function of other immune responses after ischemic stroke

In addition to the above‐mentioned immune cells that have been intensively investigated after cerebral ischemic stroke, there are also some special subsets of immune cells that gained relatively less attention in the field of poststroke BBB integrity, including mast cells, dendritic cells, B lymphocytes, and NK cells.^158^, ^159^, ^160^ B cells are important adaptive immune cells that have been suggested to have beneficial effects on the ischemic brain as early as 24‐48 hours after MCAO.^161^ Lack of B cells substantially increases infiltration of various leukocyte subpopulations into the brain and exacerbates the BBB disruption.^162^ On the other hand, B cells may also have detrimental effects to the ischemic brain injury by eliciting antibody‐mediated immune response. Since brain proteins are detected in the cerebral‐spinal fluid and the peripheral blood of stroke patients, these proteins could elicit the activation of antigen‐presenting cells, such as dendritic cells after stroke and later on even induce antibody production from B cells, just like what have been seen in multiple sclerosis lesions.^159^, ^160^ NK cells are key members of the innate immune system, accumulating in the ischemic hemisphere.^163^, ^164^ NK cells can function as very early responders to pathogen invasion through their cytolytic activity.^163^, ^164^ In mice with large infarcts induced by MCAO, NK cells promote local inflammation and exacerbated brain infarction and BBB damage and determine the size of the brain infarct.^164^, ^165^ In addition, the activation of the complement system, which is part of the innate immune response, has been described in clinical and experimental stroke.^166^ The complement system also has dual roles during the injury and recovery of ischemic stroke.^167^ It contributes to the recruitment and activation of immune cells, especially microglia, which may worsen BBB damage.^168^ It also plays a role in stroke recovery by promoting the resolution of inflammation and regeneration.^169^, ^170^ Despite the above findings, the relationship between these immune cells and the poststroke BBB integrity remains largely unknown and investigation in this regard would be of interesting and worthwhile.

**Figure 2:**
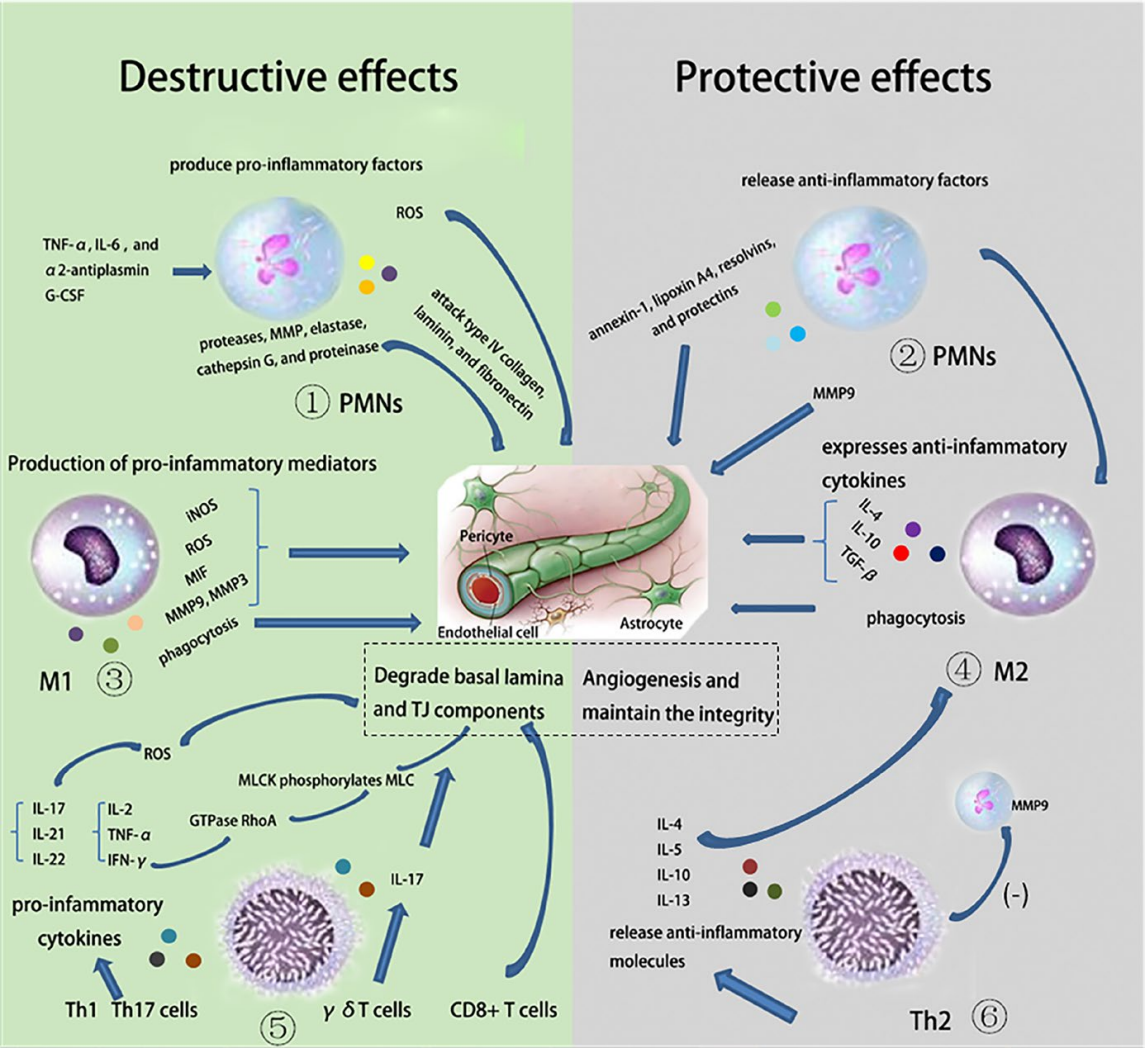
Peripheral immune cells have dual roles in poststroke BBB integrity. After ischemic stroke, the peripheral immune cells, including PMNs, macrophages, lymphocytes, infiltrate into the brain and induce inflammatory or antiinflammatory responses via distinct pathways. These responses can impact the BBB integrity in different ways. ① PMNs release proteases, MMP, elastase, cathepsin G, proteinase, and reactive oxygen species (ROS), causing endothelial dysfunction. The release of MMP9 can be induced by TNF‐α, IL‐6, α2‐antiplasmin and G‐CSF and MMP9 degrades BBB through attacking type IV collagen, lamin, and fibronectin. ② PMNs can release antiinflammatory molecules, such as annexin‐1, lipoxin A4, resolvins, and protectins to alleviate the poststroke inflammatory reaction. Neutrophil‐derived MMP9 is also involved in the regulation of pro‐angiogenesis. ③ M1 microglia/macrophages produce proinflammatory mediators including iNOS, ROS, MIF, MMP9, MMP3 et al, and phagocytize ECs, all of which induce the increase of BBB permeability. ④ M2 microglia/macrophages produce antiinflammatory cytokines and phagocytize ischemic debris to maintain the integrity of BBB. ⑤ Th1, Th17, γδT cells and CD8+ T cells have detrimental effects on BBB. Th1 cells release IL‐2, IFN‐γ, and TNF‐α, which activates the small GTPase RhoA and phosphorylates MLC then decreases TJ proteins. Th17 cells release IL‐17, IL‐21, IL‐22, and γδT cells induce IL‐17 to disrupt the BBB. ⑥ Th2 cells, especially Tregs, release antiinflammatory cytokines: IL‐4, IL‐5, IL‐10, and IL‐13, which can promote the M2 polarization. Tregs inhibit neutrophils and reduces the level of MMP9, thus protects against BBB disruption

In conclusion, the peripheral immune response is a double edge sword for the poststroke BBB. Distinct subtypes or phenotypes of immune cells may have diverse effects on the BBB disruption or repair at distinct phases after stroke (Figure 2). Understanding the roles of immune cells and their underlying mechanisms in BBB damage may help the development of promising BBB protective strategies for ischemic stroke patients.

## 4 OTHER RELEASED SIGNALING FROM PERIPHERAL IMMUNE CELLS THAT MEDIATE BBB DISRUPTION OR PROTECTION AFTER STROKE

Over the past decades, enormous efforts have been put in exploring the mechanisms underlying the BBB disruption or protection afforded by peripheral immune cells after stroke. The immune cells may impact the integrity of the BBB by direct contact of the endothelial cells, through their cell surface molecules. For example, the regulatory T cells (Tregs) are able to be recruited to the ischemic BBB through the chemokine receptor, CCR5,^153^ and inhibit neutrophil‐derived MMP9 production through the programmed death‐ligand 1 (PD‐L1) molecule, and meanwhile inhibit CCL2 expression in endothelial cells, thereby exert protective effect on BBB.^152^, ^154^, ^155^ Releasing proteins, proteinases, cytokines, and chemokines constitutes as another important mechanism to impact the BBB, in which some of the cytokines and chemokines propagate the inflammatory cascade and degrade the BBB structure as discussed above while others promote the BBB recovery (Table 1). In addition, the peripheral immune cells may also affect the BBB disruption after stroke through releasing exosomes, microvesicles, and miRNAs.

**Table 1:** Immune cell produced factors that impact blood‐brain barrier (BBB) integrity after stroke

Name	Source cells	Mechanisms	Effects	References
IL‐1	Mononuclear cells	TJ disruption; upregulation of ICAM‐1; activation of MMPs	Disruption	171, 172
IL‐6	Macrophages, T cells, endothelia cells	TJ protein loss; PKC‐dependent cytoskeletal rearrangement;	Disruption	173, 174
IL‐9	Mononuclear cells and T cells	Induce eNOS production; downregulation phosphorylated pkβ/pp3k signaling; TJ protein loss	Disruption	175, 176
IL‐17	Th17 cells and γδT cells	Induce ROS production	Disruption	34, 144
IFN‐γ	T cells	Activate the small GTPase RhoA and activate myosin light chains	Disruption	138
MIF	Endothelia cells and macrophages	Disruption TJs	Disruption	117
TNF‐α	CD4+ T cells, NK cells, neutrophils, astrocytes, and neurons	Downregulation of TJ proteins	Disruption	174, 177
CCR5	Microglia and astrocytes	Enhance MMP9 activity, regulate the migration and activity of T cells, monocytes, and dendricytes	Disruption	24, 178
CCL2	Astrocytes, microglia, EC, and macrophages	Redistribute the TJs and AJs and reorganization of actin cytoskeleton	Disruption	179, 180
HMGB1	Neurons	Induces a contractile response in pericytes and vascular ECs	Disruption	181, 182, 187
TGF‐β	Microglia/macrophages	Inhibit MMPs	Recovery	184, 185
IL‐1α	Macrophages	Induce angiogenic mediator expression and promote formation of tube‐like structures	Recovery	186, 187
IL‐10	Th2 cells	Promote the M2 polarization and maintain immune tolerance	Recovery	27, 148, 150
LCN‐2	Neutrophils and neurons	Enhance angiogenesis and induce tube formation and migration	Recovery	188, 189
HIF‐1α	Lymphocytes	Regulate VEGF and control MMPs induce BBB damage or promote angioneurogenesis	Disruption/recovery	190, 191, 192
MMP9	Neutrophils and ECs	Degrad the TJ proteins and basal lamina proteins and active proinflammatory agents: CXCL‐8, IL‐1β or TNF‐*α*	Disruption/recovery	36, 69, 89, 193, 194
		Facilitate tissue remodeling, activate bound growth factors: VEGF‐A, regulate pro‐angiogenesis		

TJ, tight junction; ICAM‐1, intercellular adhesion molecule; MMP, matrix metalloproteinase enzymes; PKC, protein kinase C; eNOS, endothelial nitric oxide synthase; ROS, reactive oxygen species; GTPase, guanosine triphosphatase; RhoA, Ras homolog gene family, member A; MIF, macrophage migration inhibitory factor; IFN‐γ, interferon gamma; TNF‐α, tumor necrosis factor alpha; NK cell, natural killer cell; CCR5, C‐C chemokine receptor type 5; CCL2, chemokine (C‐C motif) ligand 2; HMGB1, high‐mobility group box‐1 protein; TGF‐β, transforming growth factor beta; EC, endothelial cell; LCN‐2, lipocalin‐2; VEGF, vascular endothelial growth factor; HIF‐1α, hypoxia‐inducible factor‐1

### 4.1 Exosomes

Emerging evidence is showing that activation of peripheral immune cells may release exosomes and microvesicles, both of which have been implicated in the evolving of BBB damage after stroke.^195^ Exosomes are endosome‐derived small membrane vesicles. They carry proteins, lipids, and genetic materials and play essential roles in intercellular communication between source and target cells under physiological and pathophysiological conditions.^196^, ^197^ Both immune cells and nonimmune cells can secret exosomes. It is recently suggested that exosomes released from activated immune cells are responsible for carrying proinflammatory contents including miRNAs to the brain *via* the brain endothelium.^195^ These exosomes alone can activate human brain microvascular endothelial cells to increase the expression of adhesion molecules such as CCL2, ICAM1, VCAM1, and cytokines such as IL‐1β and IL‐6.^195^, ^198^ Preventing exosome release from activated monocytes could completely inhibit the expression of inflammatory molecules on brain endothelial cells and therefore regulate the BBB function under different diseases.^195^, ^198^ Exosomes from different cell types may have diverse functions on the BBB integrity. It has been shown that exosomes from circulating endothelia progenitor cells and stem cells may transfer miRNAs into cerebral endothelial cells and pericytes, thus activate PI3K/Akt signaling pathway and notch signaling pathway to mediate angiogenesis and to maintain BBB integrity.^199^, ^200^, ^201^ Thus, it is highly possible that there are specific subtypes of peripheral immune cells may release exosomes carrying BBB protective properties. However, studies in this regard are still warranted.

### 4.2 Microvesicles

Microvesicles (MVs) are small membranous vesicles released from various cells in response to diverse biochemical agents or mechanical stresses.^202^ Leukocyte‐derived microvesicles (LMVs) are one of microvesicles, which act as proinflammatory mediators implicated in some diseases.^203^, ^204^ LMVs originate from mature leukocytes, including monocyte, lymphocyte, and granulocytes.^205^ It is suggested that LMVs are involved in the vascular inflammation in cardiovascular diseases and cerebrovascular diseases including stroke.^206^, ^207^ LMVs can increase the production of TNF‐α, IL‐6, IF‐8, activated protein C, and IF‐1β^206^ and induce the translocation of NF‐kβ into the nucleus, leading to increased production of IL‐8 and monocyte chemoattractant protein 1(MCP1),^208^ both of which can promote the inflammatory response, leading to vascular endothelial cell dysfunction and vascular permeability. During cerebral ischemia, circulating MVs increase significantly and cause a large increase in barrier permeability and reduce trans‐epithelial electrical resistance (TEER) in in vitro endothelial barriers.^209^ MVs themselves contain pro‐TNF‐α, RhoA, and Rho‐associated protein kinase (ROCK), increasing the permeability of barriers in rat brain microvascular endothelial cells (RBMVECs) by activating caspase 3 and Rho/ROCK signaling pathways.^209^

### 4.3 MicroRNAs

MicroRNAs are small noncoding RNAs that broadly affect cellular and physiological function in all multicellular organisms. More than 5000 miRNAs likely exist in humans and each miRNA binds an average of 200 RNAs.^210^ MicroRNAs are divided into three categories, for example, proinflammatory, antiinflammatory, and mixed immunomodulatory. All of these regulate neuroinflammation in various pathologies, including spinal cord injury, multiple sclerosis, and ischemic stroke.^211^ After ischemic stroke, miRNAs can also mediate BBB disruption by regulating gene expression at transcriptional and posttranscriptional levels.^212^, ^213^ MiR‐130a aggravates BBB leakage and brain edema via various ways.^214^ It executes its damaging effects on BBB by downregulating HoxA5 and thereby reducing occludin expressions.^213^ Besides HoxA5, microRNA‐130a might act as a suppressor of aquaporin 4 by targeting its transcripts.^215^ MiR‐130a can also reduce the expression of caveolin‐1 and increase the level of MMP‐2/9, which contributes to the increased permeability of BBB and increased perihematomal edema after intracerebral hemorrhage.^214^ MiRNA‐15a (miR‐15a) has recently been shown to contribute to the pathogenesis of ischemic vascular injury through direct inhibition of the antiapoptotic gene bcl‐2.^216^ Of particular interest, miR‐15a itself was found to be transcriptionally regulated by peroxisome proliferator‐activated receptor (PPARδ). Administration of PPARδ agonist significantly reduced ischemia‐induced miR‐15a expression, increased bcl‐2 protein levels, and attenuated caspase‐3 activity, leading to decreased BBB disruption and reduced cerebral infarction in mice after transient focal cerebral ischemia.^216^ In addition, miR‐15a can suppress the angiogenesis in the peri‐infarct region by decreasing FGF2 and VEGF levels,^217^ thus downregulation miR‐15a can promote angiogenesis and maintain BBB integrity.^201^ Overexpression of let‐7 and miR‐98 in vitro and in vivo resulted in reduced leukocyte adhesion to and migration across endothelium, diminished expression of proinflammatory cytokines, and increased BBB tightness, attenuating barrier “leakiness” in neuroinflammation conditions.^212^ Therefore, a variety miRNAs could be used as a therapeutic tool to prevent neuroinflammation and BBB dysfunction.

Recent findings in exosomes, microvesicles, and miRNAs have evidenced that their releases from peripheral immune cells play critical roles in the evolution of BBB pathology after stroke. Notably, exosomes, microvesicles, and miRNAs released from distinct immune cells under distinct contexts may exert divergent roles on the BBB integrity after stroke.

## 5 SUMMARY AND CONCLUSION

Targeting the highly dynamic events that occur during stroke in the relatively inaccessible brain microenvironment is challenging. Emerging evidence suggests that peripheral immune cells could provide promising therapeutic targets to rescuing BBB after stroke. In clinical, some drugs with translational potential to target the peripheral immune response in order to preserve the BBB integrity after stroke are being tested in clinical settings, such as minocycline, adjudin, and curcumin.^218^, ^219^, ^220^, ^221^, ^222^ Further understanding of the interactions between the immune system and the BBB disruption and repair process could move the translation of promising preclinical results forward. Recent studies suggest that the peripheral immune response is a double edge sword both for the disruption and repair of BBB after stroke. Distinct subtypes or phenotypes of immune cells may have diverse impacts on the BBB integrity at distinct phases after stroke. Considering the double facet roles of immune cells and their pleiotropic underlying mechanisms in BBB damage and repair, we envision that researches regarding the interaction between peripheral immune cells and BBB may gain increasing attention in the pursuit of developing effective and easy accessible therapeutic targets of stroke.

## CONFLICT OF INTEREST

None.
